# The Accidental School Nurse: How Australian Registered Nurses Enter School Nursing

**DOI:** 10.1177/10598405251342700

**Published:** 2025-05-21

**Authors:** Anita Moyes, Susan Karemba

**Affiliations:** 1 School of Nursing and Midwifery, 2498 Edith Cowan University, Perth, Western Australia, Australia

**Keywords:** Australia, school nursing, schools, children, workforce

## Abstract

Australia has a long history of school nursing and a strong orientation to formal educational pathways for nursing specialization. Despite this, pathways for becoming a school nurse are lacking, and how Australian nurses became a school nurse is unclear. The aim of this study was to gain insight into this issue. Semi-structured interviews were conducted with 45 Australian school nurses. Data was analyzed using inductive content analysis. Findings identified that Australian school nurses typically became a school nurse by accident, not design. They entered school nursing having already had a long and diverse clinical career and were drawn to school nursing for distinct personal and professional reasons. A significant proportion had not encountered the specialty before becoming a school nurse. These findings emphasize a need to improve the visibility of school nursing in Australia and enhance opportunities for Australian nurses to choose school nursing as a career.

## Introduction

Over the past 50 years, the scope of nursing work undertaken in Australian schools has been characterized by a diversity of roles and activities (Moyes et al., 2025). While this provides a rich history and suggests a well-established specialty, Australian universities do not offer post-registration education for becoming a school nurse (Dunn et al., 2024). Investment in school nursing research is also minimal, but recent Australian research indicates that school stakeholders (Moyes et al., 2024) and the Australian general public (Moyes & Hayward, 2025) have a limited understanding of the scope and value of school nursing work. These findings are particularly troubling given that Australian children and young people are attending school with increasingly complex medical and mental health problems (Williams et al., 2024).

## Background

In many countries where school nursing is well established, universities and professional school nursing organizations have developed structured post-registration education and certification pathways for becoming a school nurse. In the United States, nurses seeking specialization as a school nurse undertake an examination leading to voluntary certification (National Board for Certification of School Nurses, 2025). In contrast, the United Kingdom offers a pediatric specialty at the undergraduate nursing level (National Health Service, 2025a) and subsequently the opportunity to specialize as a school nurse by undertaking postgraduate studies at master's level (National Health Service, 2025b). In Sweden, postgraduate education at the master's level is required for becoming a school nurse (Martinsson et al., 2021). Australia has no formal pathways for becoming a school nurse.

Nursing is a highly regulated profession in Australia with many opportunities for specialization. To practice as a nurse, individuals complete an accredited nursing qualification to meet requirements for registration with the Nursing and Midwifery Board of Australia (2025a). The register of qualified nurses is publicly available via the Australian Health Practitioner Regulation Agency (2025), and the title “nurse” is protected by legislation: only individuals qualified and registered can use this title (Nursing and Midwifery Board of Australia, 2025b). Despite this, recent research suggests the Australian general public is sometimes unsure whether a “school nurse” is a qualified nurse (Moyes & Hayward, 2025).

To support the demand for registered nurses, Australian universities offer pre-registration nursing education at the bachelor's degree level. Nursing education in Australian universities is highly regarded, attracting large numbers of domestic and international students (Schwartz, 2019). National standards for nursing education are regulated and monitored by the Australian Nursing and Midwifery Accreditation Council (2025). Undergraduate nursing education prepares graduates to work as beginning-level practitioners in all areas, including mental health, and this is referred to as being comprehensively registered. Information about the opportunity to specialize is introduced at the undergraduate level, and many new graduate nurses seeking specialization accept employer-funded opportunities for engaging in postgraduate nursing education shortly after their initial registration (Cleary et al., 2024).

Given the emphasis on post-registration specialization, Australian universities have invested heavily in postgraduate nursing education. A wide range of master's level courses are available, including in child and adolescent health, public health, pediatric nursing, adolescent health, youth mental health, and adolescent sexual health (Dunn et al., 2024). The sector is so large that it contributes significantly to the Australian economy (Australian Government, 2025a). Despite the long history of school nursing in Australia (Moyes et al., 2025), postgraduate education specific to becoming a school nurse is absent from the course offerings (Dunn et al., 2024). A dedicated national professional body for school nurses is also lacking. These gaps in education and professional identity create obstacles to the development of the current and future school nursing workforce.

Despite the challenges, Australian nurses continue to find ways to become a school nurse. The number of nurses working in Australian schools has not been systematically quantified, but the Australian Government's health workforce data tool suggests the number is growing (Australian Government, 2025b). Reassuringly, the Australian national school nursing standards for practice, first published in 2012, were updated in 2019 (Australian Nursing and Midwifery Federation, 2019), emphasizing the importance and requirement for these professional standards. The Australian Government is strongly focused on promoting primary health care (2025c), while the issue of poor student and teacher wellbeing (McCallum, 2021) and overwhelmed hospitals and emergency departments (Wenham & Bruce, 2024) have been acknowledged. Despite the potential of school nurses to address these intersecting issues, the published literature since 2020 reflects only two of Australia's eight states and territories (Moyes et al., 2025). Sanford and colleagues reported on the implementation of “school-based primary health care registered nurses” in an isolated rural community in the state of New South Wales (Sanford et al., 2021, 2022) and Moyes and colleagues described the work of secondary school nurses with young people experiencing mental health problems in the state of Western Australia (Moyes et al., 2022, 2023, 2024). Both roles were conceptualized as community health nursing roles: nurses were embedded into school wellbeing teams to facilitate improved access to local health services, but did not routinely manage minor student injury or illness. The lack of peer-reviewed literature reflects the lack of dedicated school nursing researchers in Australia. Not unexpectedly, the lack of literature also contributes to poor stakeholder understanding of school nursing work (Moyes & Hayward, 2025; Moyes et al., 2024). This, in turn, diminishes opportunities to secure funding for school nursing initiatives, affects the willingness of universities to offer post-registration pathways for becoming a school nurse, and contributes further to the invisibility of school nursing as a specialty in Australia.

How Australian nurses continue to become school nurses in the face of these obstacles has not been investigated. Critically, Australian nurses need to overcome the limitations posed by the absence of structured educational pathways for specialization, while simultaneously navigating the perceived low value and invisibility of school nursing work (Moyes & Hayward, 2025). The aim of this study was to explore how nurses in Australia became a school nurse.

## Methods

### Study Design

This study is the first paper from a larger program of exploratory qualitative descriptive research exploring the diversity of nursing work in Australian schools. Data was collected via semi-structured online interviews.

### Participants and Setting

Participants were required to be registered as a nurse with the Australia Health Practitioner Regulation Agency (2025) and have experience working as a nurse in an Australian school. Participants could be working in any type of Australian school, including primary and secondary schools, schools for children with special medical needs, and government and non-government schools.

### Sampling and Recruitment

Information about the study was shared to the professional networking platform LinkedIn. Regional school nursing special interest groups subsequently shared the information with their networks, resulting in a wide reach. Study information encouraged potential participants to contact the research team directly for a participant information sheet and consent form. Efforts were also made to provide government school nursing leaders in each state and territory with information about the study. Some regional school nursing leaders expressed interest in supporting the research initiative and distributed the participant information sheet and consent form to their staff. Recruitment was therefore primarily undertaken via purposive and snowball sampling (Flanagan et al., 2024). Potential participants were provided the opportunity to ask questions, and nurses who expressed interest in participating were asked to sign and return the consent form. The sample size was determined by data saturation (Flanagan et al., 2024, p. 761) across the larger qualitative study investigating the diversity of nursing work in Australian schools.

### Data Collection Method

Data was collected between December 2023 and November 2024 via Microsoft Teams at a mutually convenient time. Participants provided written consent for interviews to be recorded and automatically transcribed using Microsoft Teams. A semi-structured interview guide was employed, and the subset of data exploring how participants became a school nurse was prompted by the questions*: Please tell me how you came to be a school nurse*, and *Please tell me about your career prior to becoming a school nurse.* Limited demographic data was also collected*.* Transcriptions were reviewed and corrected for accuracy, and all potentially identifying information was removed before uploading transcriptions to NVIVO Version 12 (Lumivero, 2017) for analysis.

### Data Analysis

Data was analyzed using inductive content analysis as described by Elo and Kyngäs (2008). This approach enabled a systematic examination of the qualitative data and facilitated the development of categories that reflected participant narratives in place of using a pre-existing framework. Elo and Kyngäs (2008) outline three key steps in content analysis: i) preparation, ii) organizing, and iii) reporting.

The first step involved the preparation of the data for analysis. In this study, the researchers became familiar with the data by listening to the recorded interviews and reading the transcriptions. The second step involved organizing the data. With the aid of NVIVO (Lumivero, 2017), the researchers independently coded the sections of interview transcripts that corresponded with the relevant questions. Coding was conducted according to the methods outlined by Elo and Kyngäs (2008): the transcripts were examined line by line and phrases coded for meaning. Categories were developed exclusively from the data with an inductive approach to analysis. Similar codes were grouped into categories and compared and contrasted for similarities and differences in an iterative manner. Where indicated, categories were expanded or collapsed as data analysis progressed. Throughout the process of analysis, the researchers met regularly to discuss emerging categories and the relationship between categories.

The third and final step of the content analysis process was reporting. In this step, categories were arranged into a narrative sequence explaining how participants became a school nurse in Australia. The researchers continued to meet regularly to review the draft manuscript, discuss the findings, and confirm continuing alignment with the data analysis methods outlined by Elo and Kyngäs (2008).

### Ethical Considerations

This study received ethical approval from Edith Cowan University (REMS 2023-04848-MOYES). All participants gave informed consent before taking part in the study. Participant confidentiality was maintained by de-identifying transcripts and storing the interviews in a secure digital storage facility at the university. The study adhered to the revised Australian National Statement on Ethical Conduct in Human Research, 2025.

### Trustworthiness and Rigor

Trustworthiness was considered with reference to the work of Kyngäs et al. (2020), which specifically focuses on content analysis. To ensure credibility, efforts were made to include participants from all eight states and territories of Australia working in both government and non-government schools. Transcripts were reviewed against audio recordings to ensure accuracy. The study team consisted of a researcher experienced in school nursing and a novice nurse researcher unfamiliar with the topic, providing a diversity of perspectives. Dependability was enhanced with a systematic approach to data collection using a semi-structured interview guide, while analysis was conducted by both researchers independently, and emerging findings were compared and discussed. A clear audit trail linking the data and the findings was maintained, while the use of quotes provides rich insights and promotes confirmability, authenticity, and transferability. Finally, the COREQ checklist (Tong et al., 2007) was used to guide reporting of this study.

## Findings

### Participant Characteristics

Forty-five school nurses from every Australian state and territory participated in the study. Participants worked in both government and non-government schools, as shown in Table 1. All participants were registered nurses, two participants were male, and all but two were currently working in a school. At the time of interview, participants had between 6 years and 46 years of experience working as a nurse (mean 25.2 years) and between <1 year and 30 years of experience working as a nurse in a school (mean 7.7 years). On average, participants had worked as a nurse for 17.5 years before becoming a school nurse (range 2 to 40 years). A significant number of participants had worked in pediatrics in tertiary hospitals. Others had worked in the community, in community medical centers, in hospital-in-the-home for adults, in population health, and with the Royal Flying Doctor Service. Three participants had worked as paramedics, others had worked in child protection, family and domestic violence, drug and alcohol services, and with pregnant teenagers. One had previously worked as a teacher and another as a dental nurse. Participants also had a wide range of postgraduate qualifications, often at the master's level, and many had held senior nursing positions such as director of nursing, after-hours hospital manager, and clinical nurse manager. Some had worked as tutors at university, and others had experience in project managing funded nursing research. The interview time ranged from 18 to 48 min (mean 30.2 min).

**Table 1.** Number of Participants per Australian State/Territory and Education Sector.

Australian State or Territory	Total number of nurse participants	Participants working in non-government schools	Participants working in government schools
New South Wales	8	1	7
Queensland	11	10	1
Victoria	8	3	5
Tasmania	4	0	4
Australian Capital Territory	4	0	4
South Australia	1	1	0
Northern Territory	2	1	1
Western Australia	7	3	4
Total	45	19	26

### The Accidental School Nurse

The main category identified in the analysis was “the accidental school nurse.” This category captured the overall finding that Australian school nurses became a school nurse by accident rather than by design. Four subcategories were also identified: (i) “I didn’t plan it”; (ii) “the opportunity arose”; (iii) “it just ticked all the boxes”; and (iv) “I’ll give that a go.”

#### Sub-Category 1: I Didn’t Plan It

Most participants had undertaken their nursing training in Australia and very few set out to become a school nurse: “I think it was by accident… it certainly wasn't on my radar to become a school nurse” (P19). Some participants reported that they had not even encountered the speciality prior to becoming a school nurse, and rarely conceptualized school nursing as a specialty to be chosen. One participant even distinguished between being aware of an individual school nurse and being aware of school nursing as a specialty: “[it was] not something I'd ever looked at doing before. I don't think I even really knew [the specialty] existed other than that [my] friend was doing it” (P41). Even participants who were interested in school nursing had typically avoided forming an intention to become a school nurse. One described a long period of liking “the look of it,” another described “joking” for many years that they should become a school nurse, while a participant with more than 30 years’ experience before becoming a school nurse explained: “in the back of my mind, I was always thinking, maybe I could be a school nurse” (P16).

A minority of participants had trained in countries such as the United Kingdom (UK), which has well-established school nursing education programs, and these participants were significantly more likely to have planned a career as a school nurse:

When I was studying [in the UK] we had a research dissertation to do in our 4th year [of undergraduate studies] and my research dissertation was very focused on schools… from then on, I just had an interest in school nursing. (P24)

For many participants, becoming a school nurse had much more to do with opportunity.

#### Sub-Category 2: The Opportunity Arose

In describing the decision to become a school nurse many participants specifically used the word “opportunity,” although the opportunity perceived varied. Some noted the opportunity to use primary health care skills, others the opportunity to be autonomous or innovative in a nursing role. Other participants described circumstances that aligned with pursuing opportunity:

[I] certainly wasn't looking for a job in a school. I'd been working [elsewhere] and saw the [job advertised], and I thought, ‘ok, that's similar to what I had been doing in [another role]. I'll apply.’ Went for the interview, got the job. (P19)

Although most participants noted opportunities arose in the form of seeing a position advertised in the newspaper or online, others explained the value of personal connections in alerting them to opportunities. Some had friends working in schools who were aware of upcoming opportunities, while several participants described being in the right place at the right time:

I rang the principal at the [secondary] school one day and [said] is there anything that I can do? … helping at lunchtime activities … or starting a breakfast club or something? He told me … this job [was] coming up… Without him, I wouldn't have had any idea about the role. (P35)

A smaller number of participants were approached directly by a school because they had a particular skill or background that the school was seeking, such as being proficient in Auslan (Australian sign language) or having attended boarding school and being familiar with boarding school life. Although opportunity was instrumental, participants were significantly encouraged in their decision by the personal and professional benefits they perceived school nursing offered.

#### Sub-Category 3: It Just Ticked all the Boxes

An overwhelming number of participants explained that employment as a school nurse met their personal needs, their professional needs or both. Of these, personal needs were often the most emphasized.

A key reason for accepting employment as a school nurse was that it presented a good fit with participants’ personal lives, especially the demands of family life. Participants identified that working “school hours” was attractive. Not needing to arrange childcare around shift work and not having to work on weekends or during school holidays were also major factors in their decision to become a school nurse. Two participants noted nightshift was especially onerous and difficult. Some participants described being a single parent or having a partner who was away for long periods working “FIFO” (fly-in, fly-out). Several participants reported injury prevented them from being able to do heavy ward-based nursing work, while others wanted a less physically demanding role as they approached the latter part of their career. For many, it was a combination of factors:

I was tired of doing shift work. My kids were going into senior school, [and] I wanted to be available more in the evenings and weekends to help them. Initially [the school nurse job] was part time, but almost full time, [around] 35 hours a week. It was school terms which meant I could have the holidays off with my kids. Have time as a family. It just ticked a lot of boxes. (P13)

Other participants emphasized school nursing was professionally attractive. For some participants, school nursing was attractive because it had commonalities with previous roles and provided opportunities to be more autonomous and use their skills in a different setting. Others were motivated by their own experiences, such as having been a teenage mother or having had a difficult adolescence. For these participants, the opportunity to change health trajectories was a strong motivating factor for becoming a school nurse:

When you read client files and you go through the history of stuff and you think ‘if only somebody did something then’… ‘if only something happened there’… I wanted to be part of someone's story where they didn't have to worry about those ‘if onlys’. (P1)

Although many participants identified that they felt pulled towards school nursing, many participants also disclosed that they felt a need to leave previous roles. Some were unhappy in previous workplaces or needed to “do something different.” Participants spoke of needing a change from hospital work, feeling burnt out in previous nursing roles, or wanting to leave a health service that was not functioning well. For some participants, work that had previously felt fulfilling became less so as their personal circumstances changed. This participant had an extensive background as a pediatric intensive care (ICU) nurse:

I was always the shift coordinator or an acting nurse manager or an acting nurse educator… when I had children … I wanted to get out of acute children's nursing, because it was quite close… I [didn’t] want to see that anymore. (P10)

Although participants articulated personal and professional factors that contributed to their becoming a school nurse, the decision itself was typically articulated in a tentative manner.

#### Sub-Category 4: I’ll Give It a Go

When describing the decision to become a school nurse, participants used phrases conveying a spirit of adventure that sometimes bordered on the flippant. The phrase “I’ll give it a go” was featured frequently, indicating a willingness to try: “one night shift, I was looking at the jobs website… and I thought ‘I'll give that a go’” (P45). Other participants used similar phrases: “one of my friends… [showed me the job advert] … and I was like, ‘cool, I'll give it a crack’” (P39). Participants described throwing their “hat in the ring,” “jumping in,” and making a “left turn,” all indicating a willingness to try school nursing.

This sense of adventure was also evident in the diversity of experience participants brought. Many participants had worked overseas or in atypical settings such as remote areas, prisons, and for the Royal Flying Doctor Service. Although many participants had a pediatric background, diversity of experience was a much stronger finding. One participant described themselves as a “jack of all trades” (P33), while another explained: “I've done a lot of different things” (P15). Many participants indicated that they enjoyed variety and actively sought it out:

[I’m] a nurse who's moved around a lot. I don't think I'd ever had a permanent position… [before becoming a school nurse] … I like variety and [in school nursing] you never know what you're going to come across. (P30)

Participants often pointed out that a diversity of experience was crucial to school nursing work:

[In school nursing] you have to know… about a lot of different health issues… I've noticed that staff that’ve worked in one particular area for a long-time struggle with all the other things … because they're not things they're familiar with. (P15)

Consistent with this diversity of experience, participants often described their first foray into school nursing as a temporary commitment, either because it was a secondment, a pilot program, or covering leave for another nurse: “[It] was only really meant to be a short-term thing. And then nine years later here I am running the place” (P7). Becoming a school nurse was therefore gradual in the sense that participants did not necessarily expect to stay in the role when they first started out. And despite bringing a wide variety of experience and a sense of adventure, the learning curve was sometimes steep:

It was a massive change… Had I known … I don't know that I would have done it, but I absolutely love it now… the first 12 months to two years were a challenge. It's a very different role working in a [school]. (P13)

## Discussion

This study explored how Australian registered nurses became a school nurse. Importantly, the researchers identified nurses working in schools in every state and territory of Australia, in government and non-government schools. This is consistent with previous research describing the long history of nursing work in Australian schools (Moyes et al., 2025), but to the best of our knowledge, this is the first national study of Australian school nurses. It is also the first study to explore how Australian nurses became a school nurse, making an important contribution to school nursing knowledge in the Australian context. Our finding that Australian school nurses rarely planned to become a school nurse is novel and important because it highlights a need to improve the visibility of school nursing as a specialty and provide Australian nurses with enhanced opportunities to choose school nursing as a career.

Although the school nursing workforce has been of interest to researchers in countries such as the United States (Willgerodt et al., 2024), a review of international literature failed to identify studies investigating the initial decision to become a school nurse. Maeland and colleagues (2023) identified that Norwegian school nurses stayed in school nursing because of the “attractive scope of practice” and “varied tasks”. We identified similar factors attracting Australian nurses to school nursing. An Australian paper, now more than a decade old, investigated reasons for leaving school nursing and noted that some nurses used school nursing as a “stepping stone” to other career opportunities (Sendall et al., 2014). We did not investigate why Australian nurses leave school nursing, however, participants in our study reported that they had undertaken many roles prior to becoming a school nurse and enjoyed a diversity of clinical practice. It is possible that factors that prompted participants to try school nursing may also prompt them to seek new experiences in other specialties in the future.

The role of opportunity in becoming a school nurse is highly relevant in the Australian context. Australian nurses often make decisions about specialization in their final year of undergraduate nursing education, typically in response to university clinical placements (Anyango et al., 2024). In our study, only one participant recalled previous experience of school nursing as an undergraduate student and they undertook pre-registration training in the United Kingdom. The remaining participants did not disclose undergraduate or post-registration clinical experiences as influential in their decision to become a school nurse. Participants in this study typically had long and diverse nursing careers prior to becoming a school nurse. They brought clinical skills, knowledge, and a willingness to try school nursing, but a significant proportion had either not encountered the specialty before or had not formed a serious intent to become a school nurse. This suggests that Australian nurses are not encouraged to think of school nursing as a viable career choice, and is consistent with the limited understanding of school nursing demonstrated by the Australian general public more generally (Moyes & Hayward, 2025). Improved visibility and more effective information provision at the undergraduate and post-registration levels would enhance the likelihood of Australian nurses choosing school nursing, but the lack of formal post-registration educational pathways for becoming a school nurse poses an additional barrier.

The complexity of the school nursing role is not in doubt, and the need for post-registration education and clinical experience was affirmed by participants in our study. Children and young people in Australia are attending school with increasingly complex medical and mental health problems (Williams et al., 2024), and the Australian national standards for school nursing practice provide insight into the high degree of complexity Australian school nurses may need to navigate (Australian Nursing and Midwifery Federation, 2019). More than a decade ago, a significant reason for school nurse attrition in the Australian state of Queensland was attributed to the emotionally demanding clinical work and the ‘politics’ of working between two government departments (Sendall et al., 2014). Nurses reported high levels of emotional fatigue managing difficult clinical presentations such as suicide risk or suspected sexual abuse, while the discretionary powers of school staff led some school nurses to feel professionally disrespected and undervalued (Sendall et al., 2014). Post-basic education could prepare nurses for these challenges, but the absence of formal educational pathways for becoming a school nurse in Australia stands in contrast to countries such as the United Kingdom and Sweden that offer formal educational pathways for becoming a school nurse. It also stands in contrast to the Australian emphasis on formal postgraduate education for specialty practice (Cleary et al., 2024). Fortunately, Australian universities are already offering postgraduate nursing education with incidental relevance to school nursing (Dunn et al., 2024). This suggests a capacity to develop more relevant course offerings for domestic and international nurses seeking to specialize as a school nurse.

Finally, participants in our study identified that they became a school nurse because it was personally and professionally attractive, and we argue this finding requires further exploration. In common with many countries, Australia struggles to retain an adequate nursing workforce (Alshahrani, 2022). The decision to become a school nurse can be perceived as contributing to the loss of nurses from hospitals. There is some support for this position in the current study, as some participants identified reasons for wanting to leave hospital-based nursing. An alternative perspective is that school nursing offers new opportunities for influencing nurses to stay in nursing. Nursing remains a female-dominated workforce, and shift work continues to be challenging for many modern Australian families (Alshahrani, 2022). A key factor we identified was that participants in our study were motivated to become a school nurse because of the working hours school nursing offered. The potential benefit of retaining nurses who might otherwise be lost from the system should be explored further.

## Limitations

This study employed participant self-report. As participants had typically been employed as a school nurse for a considerable period of time, there is a risk that recall may be inaccurate. While the study sought a large and diverse sample from across the Australian states and territories, the results may not be generalizable to all school nurses in Australia or to those in other countries with different education or health systems.

## Conclusion

This study is the first national study of school nursing in Australia. We identified that Australian school nurses typically became a school nurse by accident, not design. A significant proportion of Australian school nurses had not encountered the specialty before becoming a school nurse themselves and were drawn to the specialty for distinct personal and professional reasons, typically after a diverse clinical career. These findings emphasize a need to improve the visibility of school nursing in Australia and enhance opportunities for Australian nurses to choose school nursing as a career.
